# Thyroxine (T_4_) Transfer from Blood to Cerebrospinal Fluid in Sheep Isolated Perfused Choroid Plexus: Role of Multidrug Resistance-Associated Proteins and Organic Anion Transporting Polypeptides

**DOI:** 10.3389/fneur.2017.00214

**Published:** 2017-05-23

**Authors:** Kazem Zibara, Nabil El Zein, Mirna Sabra, Mohammad Hneino, Hayat Harati, Wael Mohamed, Firas H. Kobeissy, Nouhad Kassem

**Affiliations:** ^1^ER045, PRASE, Lebanese University, Beirut, Lebanon; ^2^Faculty of Sciences, Biology Department, Lebanese University, Beirut, Lebanon; ^3^Neuroscience Research Centre, Faculty of Medical Sciences, Lebanese University, Beirut, Lebanon; ^4^Faculty of Public Health, Medical Laboratory Department, Lebanese University, Beirut, Lebanon; ^5^Basic Medical Science Department, Kulliyyah of Medicine, International Islamic University Malaysia, Kuantan, Pahang, Malaysia; ^6^Neuroscience Unit, Menoufia Medical School, Cairo, Egypt; ^7^Department of Biochemistry and Molecular Genetics, American University of Beirut, Beirut, Lebanon

**Keywords:** transport, thyroid hormone, blood–cerebrospinal fluid barrier, blood–brain barrier, efflux, uptake

## Abstract

Thyroxine (T_4_) enters the brain either directly across the blood–brain barrier (BBB) or indirectly *via* the choroid plexus (CP), which forms the blood–cerebrospinal fluid barrier (B-CSF-B). In this study, using isolated perfused CP of the sheep by single-circulation paired tracer and steady-state techniques, T4 transport mechanisms from blood into lateral ventricle CP has been characterized as the first step in the transfer across the B-CSF-B. After removal of sheep brain, the CPs were perfused with ^125^I-T_4_ and ^14^C-mannitol. Unlabeled T_4_ was applied during single tracer technique to assess the mode of maximum uptake (*U*_max_) and the net uptake (*U*_net_) on the blood side of the CP. On the other hand, in order to characterize T_4_ protein transporters, steady-state extraction of ^125^I-T_4_ was measured in presence of different inhibitors such as probenecid, verapamil, BCH, or indomethacin. Increasing the concentration of unlabeled-T_4_ resulted in a significant reduction in *U*_max_%, which was reflected by a complete inhibition of T_4_ uptake into CP. In fact, the obtained *U*_net_% decreased as the concentration of unlabeled-T_4_ increased. The addition of probenecid caused a significant inhibition of T_4_ transport, in comparison to control, reflecting the presence of a carrier mediated process at the basolateral side of the CP and the involvement of multidrug resistance-associated proteins (MRPs: MRP1 and MRP4) and organic anion transporting polypeptides (Oatp1, Oatp2, and Oatp14). Moreover, verapamil, the P-glycoprotein (P-gp) substrate, resulted in ~34% decrease in the net extraction of T_4_, indicating that MDR1 contributes to T_4_ entry into CSF. Finally, inhibition in the net extraction of T_4_ caused by BCH or indomethacin suggests, respectively, a role for amino acid “L” system and MRP1/Oatp1 in mediating T_4_ transfer. The presence of a carrier-mediated transport mechanism for cellular uptake on the basolateral membrane of the CP, mainly P-gp and Oatp2, would account for the efficient T_4_ transport from blood to CSF. The current study highlights a carrier-mediated transport mechanism for T4 movement from blood to brain at the basolateral side of B-CSF-B/CP, as an alternative route to BBB.

## Introduction

Thyroid hormones (THs) are important regulators of normal growth and development in the central nervous system (CNS) and brain (1–4). Thyroxine (T_4_), a major type of lipophilic TH, is transported between blood and cerebrospinal fluid (CSF) in a restricted manner, which does not follow simple diffusion mechanism (5, 6). The absence of triiodothyronine (T_3_) and thyroxine (T_4_) hormones, such as in hypothyroidism, leads to serious damage in the brain and neuronal cells (7). Therefore, it has been suggested that the blood–brain barrier (BBB) and/or blood–CSF barrier (B-CSF-B) control thyroxine availability to the cerebral compartments (8). Indeed, thyroxine enters the CSF and brain parenchyma by two possible routes: either across the BBB, located at the level of cerebral capillary endothelium into brain extracellular fluid (ECF) and then by diffusion into the CSF (9), or *via* the B-CSF-B, formed by the choroid plexus (CP) epithelium (9, 10). However, the quantitative extent to which the BBB and B-CSF-B/CP contribute to T_4_ transport to the brain is poorly understood.

It was previously shown that the level of THs increases rapidly, within minutes of their intravenous (*i.v*.) injection, using an *in vivo* dog model (11). In fact, T_3_ cross instantly from blood into CSF through a carrier-mediated process. In addition, it was demonstrated that 1 h after *i.v*. injection of radiolabeled-T_3_, it accumulates to a large extent in the CP and gray matter, before any appearance in the white matter of the brain (12). This accumulation in the CPs, and the subsequent rise in the CSF levels, cannot be accounted for through a free diffusion mechanism from circulating plasma where THs are mostly found as protein bound. However, this increase is likely to occur through a carrier transport mechanism present at the blood side of the CP. Since the rate of T_4_ equilibrium into CSF is more rapid than that into brain, the CP might constitute another major pathway for the entry of these hormones into the CSF.

Thyroid hormone action at the cellular level depends primarily on the binding of T_3_ to its nuclear receptors (13), through type 2 deiodinases (D2), expressed in astrocytes. In fact, ~50% of intracellular T_3_, active form of the hormone, derives from T_4_ already present within the brain. On the other hand, the remaining 50% of T_3_ depends on the entry of T_4_ from the circulation into the brain through various transporters that act across the BBB and CP. However, the transport mechanisms of THs into brain and the role of the CP transporters in this context are still poorly understood.

Using a rat model, a carrier transport mechanism was identified for T_3_ and T_4_ uptake at the BBB; however, their high accumulation by the CP was not investigated (14). Furthermore, it was shown that CP of the rat can accumulate T_4_ more rapidly than any other region in the brain (15). We have also revealed in an *in vivo* rabbit model that the distribution of T_4_ from CSF into the brain and CP is dependent on carrier-mediated transport mechanisms (16). In fact, the CP may potentially contribute to THs homeostasis in the brain ECF since the CSF secreted by the CP is in direct contact with the ventricular/sub-ventricular regions and the brain interstitial fluid (ISF). On the other hand, the BBB has been thought to be the major pathway for T_3_ and T_4_ entry into CNS ISF since its surface area is greater than that of the CP. Nevertheless, it was shown that the surface area of the CSF face of the CP may have a greater transport capacity, especially during early stages of brain growth and development (17).

Different transport mechanisms for the movement of THs from CP epithelial cells into CSF (15, 18) and from CSF into brain (19, 20) have been identified in earlier studies. However, only limited information is known about the initial uptake process from blood to CP and then into CSF. Several limitations exist for the study of T_4_ uptake using *in vitro* and *in vivo* techniques. In fact, *in vitro* studies are complicated by the inability to gain access to the blood side of the CP, while *in vivo* studies cannot distinguish the transport across the B-CSF-B/CP from that across the BBB. The current knowledge on how the brain regulates TH homeostasis is incomplete, and the role of B-CSF-B/CP is still not fully understood.

In this study, we have used an *in situ*-isolated perfused CP of the sheep which can selectively examine the B-CSF-B/CP, in complete separation from the BBB (19). Indeed, we have previously demonstrated, using this model, that ^125^I-T_3_ uptake at the blood face of the CP was mediated by both saturable and non-saturable uptake processes (19). Therefore, this study investigates the extraction of ^125^I-T_4_ at the basolateral (blood) side of the *in situ* perfused CP of the sheep, and the role of some protein transporters. Finally, the characteristics of T_4_ transport mechanisms were also examined using various drug inhibitors.

## Results

### CSF Secretion Rate

**Figure 1:**
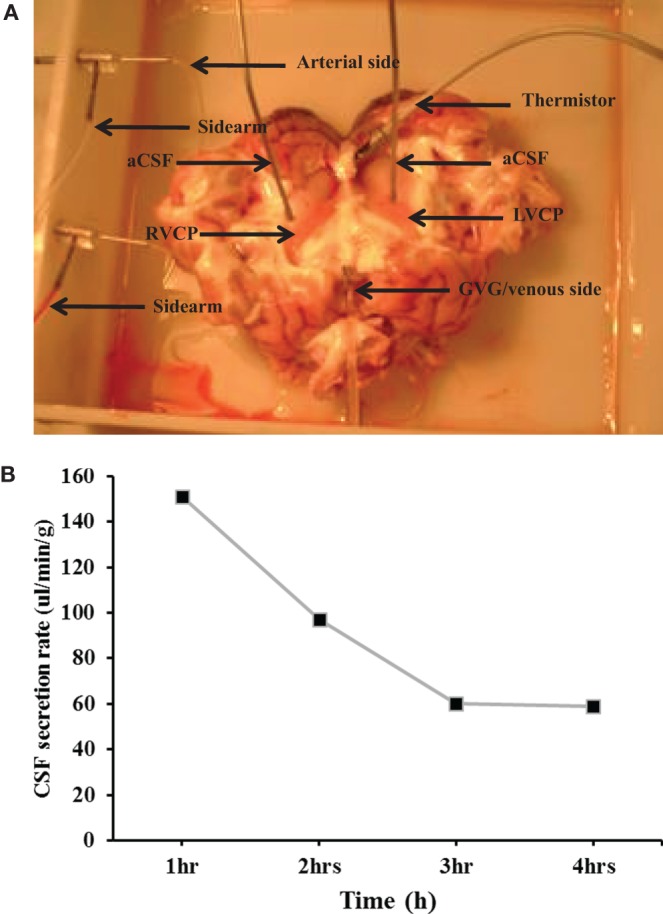
**(A)** The *in situ*-perfused choroid plexus (CP) of the sheep model. LVCP, left ventricle choroid plexus; RVCP, right ventricle CP; aCSF, artificial cerebrospinal fluid; GvG, great vein of Galen. **(B)** The average cerebrospinal fluid (CSF) secretion rate. The rate of CSF secretion was performed during the 4 h of CP perfusion (*n* = 14).

The CSF secretion rate was measured in an *in situ*-isolated perfused CP of the sheep (Figure 1A). Experiments were stopped after 4 h of perfusion since an increase in the arterial pressure and a decrease in the CSF secretion rate were observed, clear indications of tissue deterioration. Results showed that the rate of CSF secretion remained constant during the 4 h of CP perfusion. In fact, the average secretion rate during 4 h was 132.1 ± 4.4 µl/min/g (*n* = 14, Figure 1B), consistent with previously published studies (21).

### Uptake of ^125^I-Labeled T_4_ Using Single Circulation Method

**Figure 2:**
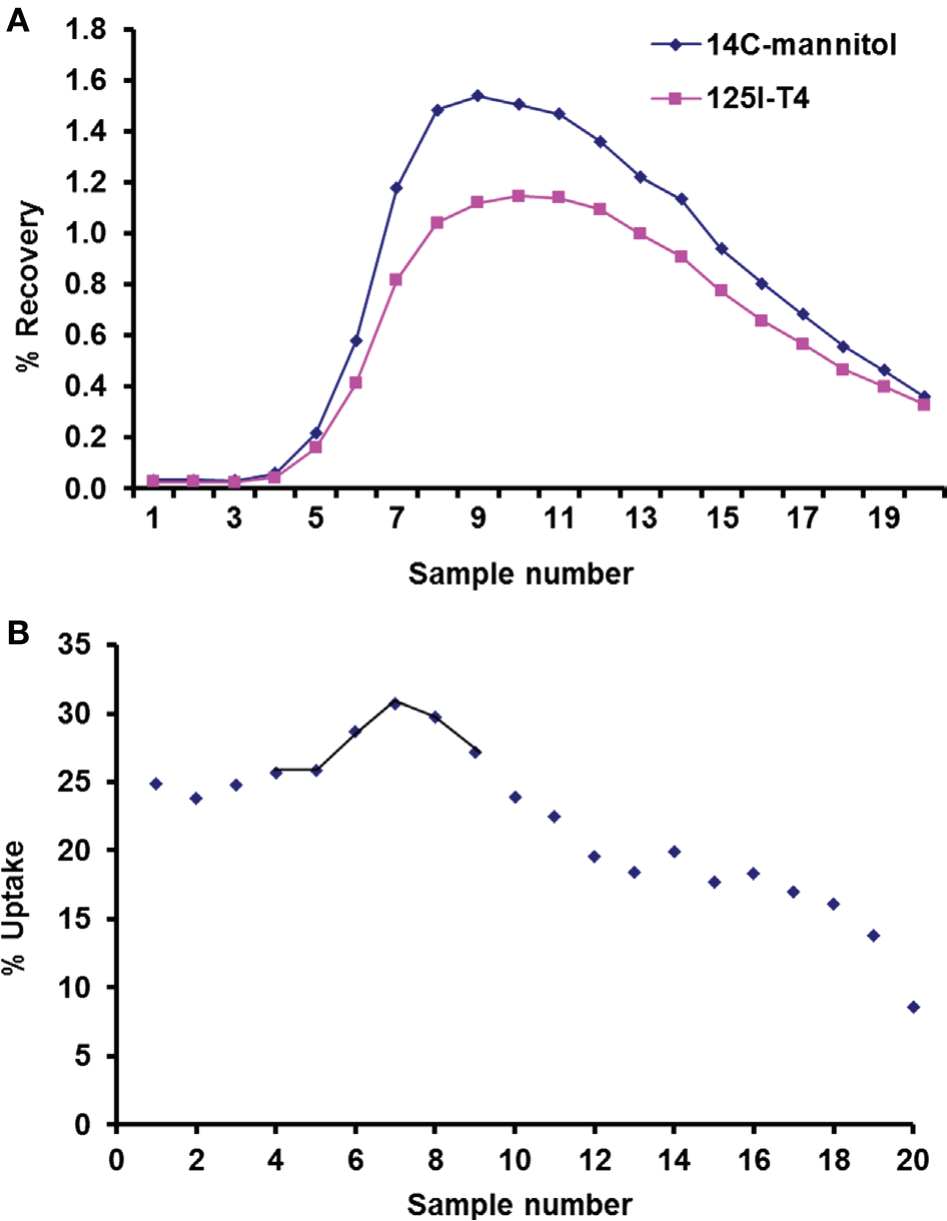
**Uptake of ^125^I-labeled T_4_ using single circulation method**. **(A)** Recovery of ^14^C-mannitol and ^125^I-T_4_ in a representative run of 20 consecutive venous samples, plotted as a percentage (%) of radioactivity injected in the 100-µl bolus. The lower recovery curve of ^125^I-T_4_ relative to ^14^C-mannitol indicates T_4_ uptake at the basolateral face of the isolated perfused choroid plexus (CP). **(B)** Uptake (%) of ^125^I-T_4_ in each venous sample relative to ^14^C-mannitol plotted against the sample number. Samples that contained the greatest recovery of isotopes are joined by a line, which were averaged to estimate the *U*_max_ (%) at the basolateral side of the isolated perfused CP.

Mannitol was used as an extracellular marker, which allowed the measurement of thyroxine net cellular uptake, using the single-circulation paired tracer dilution technique at the basolateral side of the isolated perfused CP (Figure 2A). Indeed, mannitol can only diffuse from the vascular compartments across the fenestrated capillaries and is not taken up into the CP cells *via* any carrier-mediated process (10, 19). However, some mannitol diffuses across the CP *via* the paracellular route as the CP tight junctions are more permeable than those of the BBB. Comparison between the percentage recoveries of thyroxine versus that of mannitol (Figure 2A) enables the measurement of the net cellular uptake across the plexuses (Figure 2B) and hence corrects for any diffusion between the cells. Results showed that during the first 10 s of perfusion, the average maximum uptake of ^125^I-labeled T_4_ on the blood side of the CP was found to be 30% (Figure 2B).

**Figure 3:**
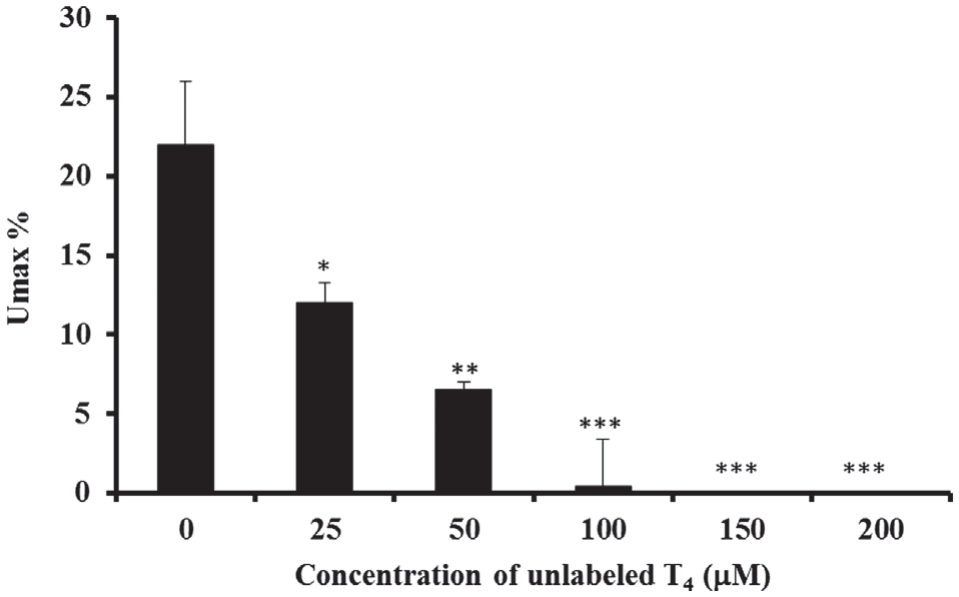
**Calculation of *U*_max_ and *U*_net_**. The inhibitory effect of different concentrations of unlabeled-T_4_ on the calculated *U*_max_% in the isolated perfused CP of the sheep, using the single-pass method. The maximum uptake of T_4_ (*U*_max_) is recorded when the maximum uptake of radioactivity has occurred. Results are expressed as the mean ± SEM. Statistical significance were determined using the Student’s *t*-test and shown as *p < 0.05, **p < 0.001, ***p < 0.0001.

The characteristics of basolateral transport of ^125^I-labeled T_4_ were then investigated, by measuring the maximum uptake (*U*_max_) in 20 drops of perfusate and in less than 60 s time period (Figure 3). Results showed that there was a significant decrease in the *U*_max_% in presence of different concentrations of unlabeled-T_4_ (Figure 3). Indeed, the *U*_max_% fell from ~22%, when only trace levels of ^125^I-labeled T_4_ were present, to ~12% after the addition of 25 µM unlabeled-T_4_ (Figure 3). In addition, a higher concentration of unlabeled-T_4_ (50 µM) caused a further significant reduction in the *U*_max_%, indicating increased saturation of T4 carrier-mediated proteins. Moreover, complete saturation was achieved in presence of 100 µM of unlabeled T_4_ (Figure 3).

Furthermore, data also showed that the obtained *U*_net_% decreased as the concentration of unlabeled-T_4_ increased (Table 1). The inhibitory effect on *U*_net_% ranged between ~45 and 71%, which was consistently lower than *U*_max_, suggesting the presence of a significant amount of tracer backflux with time (Table 1).

**Table 1:** **The effect of different concentrations of unlabeled thyroxine (T_4_) on the net uptake (*U*_net_%) of radiolabeled ^125^I-T_4_ from the blood side of the isolated perfused choroid plexuses of the sheep, using the paired tracer dilution technique**.

	*U*_net_ (%)	Inhibition (%)
Control (1.8 nM)	17.0 ± 2.6	n.d.
25 µM	9.1 ± 1.5*	44.7
50 µM	2.1 ± 0.2***	71.2
100 µM	0***	100
200 µM	0***	100

The inhibitory effect is represented in percentages (%). Values correspond to mean ± SEM, number of sheep *n* = 4 for each condition. *p < 0.05, ***p < 0.001, in comparison to control.

In summary, increasing the concentration of unlabeled-T_4_, from 25 to 200 µM, resulted in a significant reduction in *U*_max_%, which was reflected at various levels of inhibition. In fact, high concentrations of 100 and 200 µM of unlabeled-T_4_ caused a complete inhibition of T_4_ uptake into CP. Taken together, there was a significant decrease in the *U*_max_% in presence of different concentrations of unlabeled-T_4_, consistent with an increase in the inhibition level.

### Effect of Various Drugs on the Extraction of ^125^I-T_4_, Using the Steady-state Method

**Figure 4:**
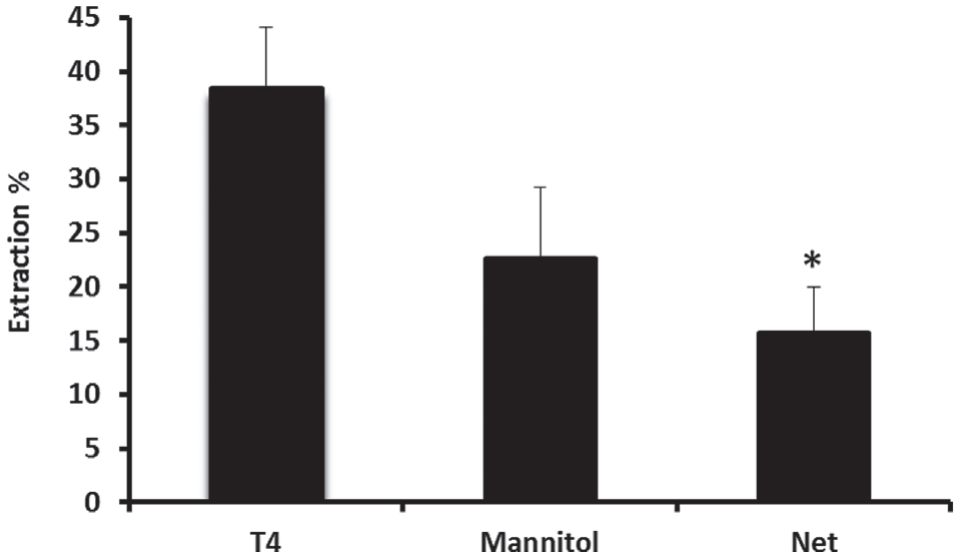
**Net extraction of T4 using the steady-state method**. Steady-state extraction of ^125^I-labeled T_4_ from the blood side was ~38%, whereas the net extraction reached ~16%, when the reference molecule mannitol was subtracted. Results are expressed as the mean ± SEM. Statistical significance were determined using the Student’s *t*-test and shown as *p < 0.05.

The characteristics of basolateral transport of thyroxine under the effect of various drugs were then investigated using the steady-state method. However, before evaluating the effect of each drug on the uptake of ^125^I-labeled T_4_, steady-state extraction uptake of ^125^I-labeled T_4_ was performed by collecting perfusates every 4 min, for a period of 1 h (Figure 4). In the steady-state method, the perfusion fluid contained 0.555 MBq of ^125^I-T_4_ tracer and 2.77 MBq of ^14^C-mannitol extracellular marker in 100-ml perfusate. Results showed that steady-state extraction of ^125^I-labeled T_4_ from the blood side was ~38% (Figure 4). In addition, the net extraction of ^125^I-T_4_ reached ~16%, when the reference molecule mannitol was subtracted, indicating a role for protein transporters on the blood side of the tissue.

In order to understand the mechanism by which T_4_ is transported from the blood to CSF across the CP, a number of drugs were used to target multidrug resistance-associated proteins (MRPs: MRP1 and MRP4), organic anion transporters and P-glycoprotein (P-gp). It has been suggested that these transporters might be involved in the uptake of thyroxine from blood into CP (20–23). The steady-state extraction of ^125^I-T_4_ at the blood side (basolateral) of the perfused CP over 1 h was calculated relative to the extracellular marker ^14^C-mannitol. This allows to measure the extraction of ^125^I-T_4_ in presence of drugs known to inhibit the efflux transporters, at the basolateral side of the CP, such as probenecid, verapamil, BCH, and indomethacin (24, 25). The net extraction of ^125^I-T_4_ from the blood to CP, which represents the non-specific paracellular loss of T_4_, was calculated by subtracting the extraction of^14^ C-mannitol from that of ^125^I-T_4_ (Ex.^125^_I-T4_ − Ex.^14^C_mann_). It is important to note that each drug was applied at the same concentration at the blood and CSF sides of the CP (basolateral versus apical sides, respectively).

Data showed that probenecid caused the highest percentage change, in comparison to control. Indeed, after its addition, a ~45% significant inhibition was observed in the net extraction of ^125^I-T_4_ (*p < 0.05; Table 2). This suggests that probenecid has competed with the same Oatps for T_4_ transport across the basolateral membrane of the CP. In fact, since all Oatps are probenecid sensitive (24), Oatps localized on the basolateral membrane of the CP (Oatp2 and Oatp14) (24, 26) as well as Oatp1 on the apical side (27) could mediate the transport of T_4_ from the blood to the CSF.

**Table 2:** **The effect of various drug inhibitors on the extraction of ^125^I-T_4_ from the blood side of the CPs**.

	Extraction of ^125^I-T_4_ from blood to CP (%)			
	^125^I-T_4_	Mannitol	Net Ex	% of paired control
Mean control	0.53 ± 0.03	0.32 ± 0.01	0.20 ± 0.02	n.d.
PROB (1.0 mM)	0.38 ± 0.05	0.24 ± 0.10	0.13 ± 0.05*	45.40
VERAP (10 mM)	0.45 ± 0.02	0.29 ± 0.01	0.16 ± 0.002*	34.30
BCH (5.0 mM)	0.49 ± 0.01	0.36 ± 0.02	0.13 ± 0.03*	17.60
INDO (1.0 mM)	0.41 ± 0.06	0.28 ± 0.02	0.13 ± 0.04*	16.20

This group of experiments was paired experiments, each with its own control. PROB, probenecid; VERAP, verapamil; BCH, β-2-aminobicyclo-(2,2.1)-heptane-2-carboxylic acid; INDO, indomethacin; CP, choroid plexus. Values are mean ± SEM, *n* = 3–6, student paired *t*-test. *p < 0.05, in comparison to control.

Similarly, the addition of verapamil, the P-gp substrate, at a concentration of 10 µM resulted in a significant ~34% decrease in the net extraction of ^125^I-T_4_ (*p < 0.05; Table 2). However, the addition of the large neutral amino acid analog BCH, which is specific to “L” system, has produced a modest ~17% reduction in the net extraction of ^125^I-T_4_, in comparison to control (*p < 0.05; Table 2). Finally, indomethacin, an inhibitor of the organic anion transporter 1 (Oatp1), had a similar inhibitory effect to BCH (*p < 0.05; Table 2). These results suggest that verapamil, BCH, and indomethacin reduce the net extraction of ^125^I-T_4_ indicating a role for P-gp, “L” system and Oatp1, respectively, in transporting T_4_ across the basolateral membrane, from the blood to CP.

## Discussion

The present study investigated the steady-state extraction of ^125^I-T_4_ transport at the basolateral (blood) side of isolated *in situ*-perfused CP of the sheep, in the presence of extracellular marker ^14^C-mannitol. Results demonstrated a carrier-mediated transport mechanism for T_4_ movement at the basolateral side of the CP involving various transporters. The following lines of evidence support the above statement: (1) the average secretion rate during 4 h was 132.1 ± 4.4 µl/min/g, consistent with previously published studies. (2) The average maximum uptake of ^125^I-T_4_ on the blood side of the CP was found to be 30%. (3) Increasing the concentration of unlabeled-T_4_ resulted in a significant reduction in *U*_max_%, which was reflected by a complete inhibition of T_4_ uptake into CP at concentrations higher than 100 µM. (4) The obtained *U*_net_% decreased as the concentration of unlabeled-T_4_ increased. (5) Steady-state extraction of ^125^I-T_4_ from the blood side was ~38%. (6) Using the steady-state method, probenecid caused the highest % change, in comparison to control, indicating that Oatps and MRPs localized on the basolateral membrane of the CP (Oatp2, Oatp14, MRP1, and MRP4) mediate the transport of T_4_ from the blood to CSF. (7) Verapamil, the P-gp substrate, resulted in ~34% decrease in the net extraction of ^125^I-T_4_. (8) The addition of BCH, specific to “L” system transporter, produced a modest ~17% reduction in the net extraction of ^125^I-T_4_. (9) Finally, indomethacin, an inhibitor of the organic anion transporter 1 (Oatp1), had a similar inhibitory effect to BCH.

In this study, the maximum uptake (*U*_max_) of ^125^I-T_4_ in the single pass technique was measured relative to mannitol, a passively distributed molecule. Therefore, the net steady-state extraction reflects both uptake and efflux back to the blood, resulting in a lower extraction compared to *U*_max_. Since *U*_max_ is an index of unidirectional uptake at the blood side of CP, our results suggest that the basolateral membrane has a high transfer rate for T_4_. The net uptake (*U*_net_) was more consistent than *U*_max_, which reflects a small backflux of T_4_ into the venous effluent. Our findings are consistent with a previous study showing that *U*_max_ for T_3_ was significantly inhibited at high concentrations of unlabeled-T_4_, exceeding those of physiological conditions (19). In addition, a carrier transport mechanism for T_3_ and T_4_ has also been identified at the BBB in a rat model (14). Furthermore, our previous studies have shown that in an *in vivo Ventriculo-Cisternal* perfused rabbit model, a large accumulation of ^125^I-T_4_ in the CP was reduced by 80% in the presence of 200 µM of unlabeled-T_4_ and proved to be a component of saturation (16). Our results demonstrated that the entry of T_4_ from the blood into CP is partially mediated by a saturable process, since the uptake of T_4_ was markedly inhibited by excess of unlabeled-T_4_. This suggests that a carrier-mediated transporter is localized in the CP, used as a pathway for T_4_ entry from blood into the CSF compartment. Indeed, this confirms our hypothesis that carrier-mediated transport for T_4_ at the basolateral membrane of the CP may contribute to TH homeostasis in brain ECF. Although this study did not investigate the fate of T_4_ after its entry into the CP, T_4_ is known to bind to other proteins such as albumin (Alb), thyroid-binding globulin, or transthyretin (TTR). Finally, T_4_ could also be transported into the CSF space from the CP when complexed to TTR, or as a free hormone (28).

The existence of mechanisms regulating the transport of TH has been suggested in cerebrocortical neurons (29), astrocytes (30), glial cells (31), hepatocytes (32, 33), erythrocytes (34), and skeletal muscle (35). The cellular influx and efflux of THs are facilitated by transmembrane protein transporters; therefore, this study investigated the role of some of these transporters located at the basolateral side, using the isolated perfused CP. The steady state of T_4_ at the basolateral face was measured in presence of various inhibitors such as probenecid, verapamil, BCH, and indomethacin, which were added to the blood side of CP. In steady state, the CP secretes CSF into lateral ventricle, supporting the net flux of T_4_ from blood to CSF. Since unbound T_4_ concentration in the CSF (70 pM) is much higher than that of the plasma (20 pM), the net flux cannot be determined only by the rate of CSF secretion.

In order to characterize the systems implicated in the transport of ^125^I-T_4_ from basal to apical sides of the CP, the role of organic anion transporters were examined. Following the addition of probenecid, there was a significant inhibition in the net extraction of T_4_ after >30 min of perfusion. Reduction in the net uptake of ^125^I-T_4_ is indicative of probenecid inhibition to Oatp2, a sodium-independent transporter located at the basolateral side of the CP (36). In addition, Oatp2 is also localized at the abluminal and luminal sides of the brain capillary endothelial cells and is involved in transporting anions such as taurocholate, cholate, bile acids, estrogen conjugates, ouabain, and digoxin (37). In fact, taurocholate has been shown to have a similar effect to probenecid, providing additional evidence for the role of Oapt2 in the efflux of T_4_ from brain to the blood side (38). Moreover, our data suggest that Oatp2 and Oatp3 are localized on both sides of the CP, mediating the uptake of T_4_. This is supported by a previous study in Xenopus oocytes, which showed that both transporters are multifunctional and involved in the transport of THs in the brain (39), retina, kidney, and liver (22). However, this does not exclude a role for Oatp1 or Oatp3 on the apical face of the CP epithelial cells, which are also probenecid sensitive (37, 40). Finally, Oatp14 (known as Oatp1c1) transporter has also been described to play a role in the uptake of T4 at the basolateral side of CP epithelial cells (26), and it is important in transporting T_4_ at the BBB since it has a high Km for T_4_ (41). Indeed, Oatp14 has been shown to localize on the BBB and to contribute to T_4_ uptake into brain (41). In summary, our results demonstrated that various Oatps are involved in the uptake of T_4_ from the blood into CP, and that their role in the transport of T_4_ in previous work has been underestimated.

Following the addition of verapamil, a well-established substrate for P-gp (42), a marked reduction was observed in the net extraction of ^125^I-T_4_ suggesting that P-gp multidrug resistance MDR1 is involved in T_4_ transport from blood to the CP. This clearly indicates that verapamil interacts with P-gp resulting in a reduction in the amount of T_4_ recovered from the blood side, which may then cross into CSF through the apical membrane of the plexus. In fact, previous studies showed that MDR1 localizes sub-apically of the CP and confers an apical-to-basal transepithelial permeable barrier (43). In addition, verapamil has also been demonstrated to inhibit MDR/P-gp and to slow T3 efflux from rat hepatoma, cardiomyocytes, and fibroblasts (44, 45). This is in accordance with previous data showing that verapamil inhibited the efflux of T_4_, reflecting an involvement of ABC transporter (44). Moreover, verapamil might also interact with MRP1 located at the basolateral side and therefore prevents T_4_ from exiting the CP toward the blood. Furthermore, verapamil has also been shown to reduce the unidirectional transport of the anticancer drug vincristine, from basolateral to apical side of the brain capillary endothelial cells (42).

Few studies were performed on the role of “L” system transporter in the transport of T_4_ from the blood to CP. Following the addition of BCH, an amino acid analog, a significant inhibition was observed in the extraction of ^125^I-T_4_ at the basolateral face of the CP. This suggests that removal of T_4_ from CP to the blood side is mediated by the amino acid “L” system on the basolateral side. This would ultimately affect T_4_ action within tissue cells, leading subsequently to changes in the total concentration of T_4_ available in the CSF under normal physiological conditions. Previous studies have shown that cross-competition exists between BCH and THs in mouse neuroblastoma cells (46), also reported in the BBB (47). It has also been demonstrated that BCH caused a weak inhibition of T_3_ uptake at the basolateral side, confirming “L” system contribution of TH transport in isolated perfused CP (19). Finally, it is not known whether BCH would specifically displace THs from intracellular binding sites since it does not affect cytosol–nucleus movement of T_3_ in BeWo cells (48).

Following the addition of indomethacin, an established inhibitor of MRP1 (49) and Oatp1 (50), the net extraction from blood to CP at the basolateral side was significantly inhibited. Our data suggest that MRP1 and MRP4, located on the basolateral membrane of CPs (51), and Oatp1, located at the apical side of the CPs, are involved in mediating T_4_ transport from blood to CP. Therefore, it would be expected that T_4_ accumulated in the CP since it was not transported out into CSF, which tends to oppose any further entry of T_4_ from blood to CP.

**Figure 5:**
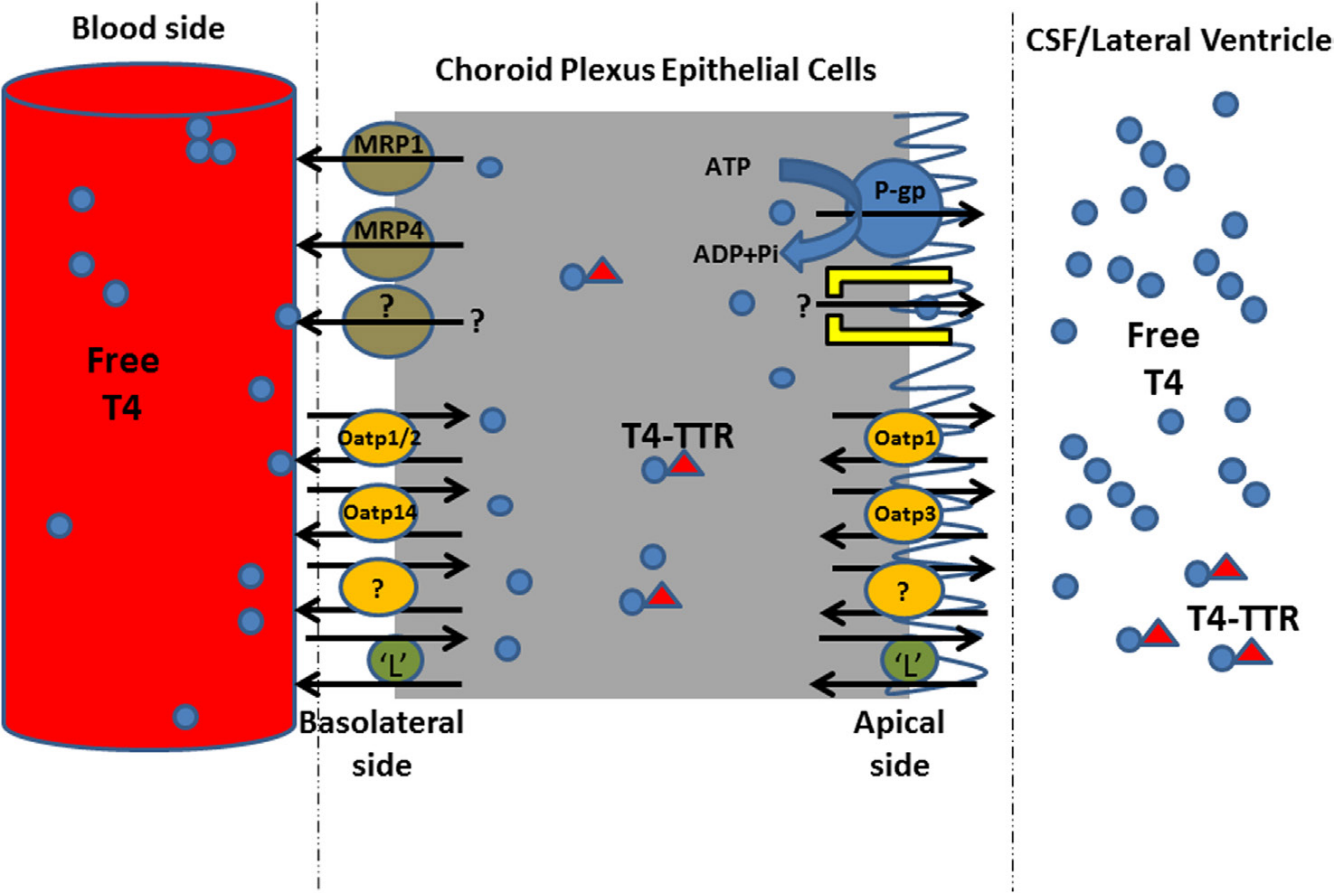
**Proposed model for T4 uptake mechanism from blood to cerebrospinal fluid (CSF) across left ventricle choroid plexus**. This schematic diagram shows the transporters of T_4_ on both sides of CPECs. T_4_ in the blood enters CPECs by carrier-mediated transporter proteins involving Oatp1, 2, and 14, and “L” system amino acid. In order to maintain T_4_ concentration in CSF/brain, P-gp and “L” system maintain T_4_ concentration in the CSF compartment, presumably during inhibition in T_4_ uptake from blood into CP. MRP, multidrug resistance-associated proteins; Oatp, organic anion transporting polypeptide; P-gp, P-glycoprotein; L, L system amino acid; TTR, transthyretin.

Taken together, the inhibition in the uptake of ^125^I-T_4_ suggests the presence of a carrier mediated process at the basolateral side of the left ventricle choroid plexus (LVCP). The presence of this carrier at the B-CSF-B may contribute to T_4_ homeostasis in the brain ECF. In addition, the significant inhibition in ^125^I-T_4_ transport in presence of probenecid suggests the involvement of Oatp1, Oatp2, and Oatp14 in the transport of T_4_ into brain. The inhibitory effect of verapamil on the extraction of ^125^I-T_4_ from blood suggests that MDR1 contributes to ^125^I-T_4_ entry into CSF. Finally, inhibition in the net extraction of T_4_ caused by BCH or indomethacin suggests, respectively, a role for amino acid “L” system and MRP1 in mediating T_4_ entry into CSF. Indeed, the carrier-mediated mechanism together with MDR1 and Oatps (Oatp1, Oatp2, Oatp14) might mediate a bidirectional transport of T_4_ from the circulating blood into the brain, playing a role in maintaining T_4_ concentration in the brain (Figure 5).

Several classes of TH transmembrane proteins belonging to the family of solute carrier (Slc) transporters have been identified such as Oatps and L-type amino acid transporters, which actively participate in the entry and exit of THs into and out of cells (52, 53). The apparent competition between the drugs that were used and the presumed T_4_ transporters, on either side of the CP, is indicative to the potential role of these transporters in T_4_ homeostasis into CP/CSF/brain. We hypothesize that the entry of T_4_ into CP tissue is not only driven by the lipid partitioning of the molecule into CP but also by a carrier-mediated transport mechanism. The drugs caused a reduction in this extraction leading to an inhibition of the transporters located on the blood side (namely, Oatp1, Oatp3, Oatp14, L system), preventing T_4_ entry into CP. Concurrently, since the latter transporters act in a bidirectional fashion, T_4_ transport toward the CSF cannot be ignored because apically localized transporters (namely, P-gp, Oatp1, “L” system) become activated, allowing T_4_ entry from CP into CSF (Figure 5). However, the role of efflux transporters MRP1 and MRP4 is to function unidirectionally, from CP to blood, in order to prevent the accumulation of T4 into CP, by removing T_4_ from CP ECs. This will allow to maintain an uphill concentration gradient of T4 in blood, balancing T_4_ concentration in CP/CSF. Since P-gps and MRPs have been reported to transport different structurally and functionally unrelated toxic xenobiotics, natural product drugs, phospholipids, and conjugated compounds (54, 55), we propose that they might directly contribute to the B-CSF barrier to the entry of T_4_ in CSF/brain. Indeed, transporters such as Lat1, Mrp1, and Mrp4 were detected on the basolateral surface of LVCP ECs (51). When the extraction was measured from blood to CP, the influx of T_4_ into the CSF was mediated *via* P-gp, whereas Oatps (Oatp1, Oatp3, Oatp14) can function bi-directionally, allowing the influx and efflux of T_4_ movement into and out of CSF. Therefore, the involvement of the latter transporters in the net flux of T_4_ from blood to the CSF might explain why the free T_4_ concentration in the CSF is greater than that found in the plasma (11). On the other hand, various substrates were reported to compete and to be transported by Mrp1, including both organic anions and some cationic compounds. For instance, glucuronide conjugates, such as estradiol-17β-glucuronide (E217βG), and sulfate conjugates, such as estrone 3-sulfate, are among those preferred substrates (56). In addition, along with GSH, Mrp1 is capable of co-transporting certain cationic compounds such as the anti-cancer drugs etoposide and vincristine (56). However, Mrp1 basolateral localization allows for efflux of substances from CSF into blood circulation (57). It is important to note that several classes of transporters such as Oatps, Na(+)/taurocholate co-transporting polypeptide, and amino acid transporters have been reported to transport TH (58).

In conclusion, the presence of transporters for cellular influx on the basolateral membrane of the CP would account for the efficient transcellular transport of T_4_ from blood to CSF, across the CP. The transport of T_4_ across the plasma membrane determines the intracellular concentration of the genomically active T_3_ (nuclear T_3_ receptor) which in turn depends on TH (T_3_ and T_4_) transport to the target cell and the activity of the different deiodinases.

## Materials and Methods

### Experimental Setup

The method of isolated perfused lateral CP of the sheep was used in this study (18, 19). Briefly, sheep of either sex (Clun Forest strain) weighing 20–35 kg and aged 6–12 months old were used. They were anesthetized with intravenous (*i.v*.) injection of thiopentone sodium (20 mg kg^−1^), heparinized (25,000 U, *i.v*.), and then exsanguinated. The brain was rapidly and carefully removed from the skull after all vessels and connections had been severed. The total number of animals used was 14 sheep. This study was approved by the ethical committee at the university.

### Cannulation of the Internal Carotid Arteries (ICAs)

Both ICAs were cannulated on the base of the brain. Perfusion system was then started at 0.5–1.5 ml⋅min^−1^ using a peristaltic pump (Watson-Marlows, UK). All other vessels in the circle of Willis were tied off in order to direct the perfusate into the anterior choroidal arteries supplying the lateral CP. The optic nerves were then sectioned allowing the brain to be removed from the skull cavity. The lateral ventricles were then opened, and the CPs were exposed and superfused with artificial cerebrospinal fluid (aCSF) and kept moist during the experiment. The venous outflow from both CPs was collected at a regular interval *via* a cannula inserted into the great vein of Galen.

### Perfusion Fluids

After cannulation of ICAs, the CPs were perfused with mammalian Ringer solution containing 4.0 g⋅dl^−1^ bovine serum albumin (Sigma Fraction V, UK). The composition of the perfusion fluids in the perfusate was (in millimolar): Na^+^ 145.8, K^+^ 5.4, Cl^−^ 119.7, ${\text{HCO}}_{3}^{-}$ 25, ${\text{HPO}}_{4}^{-}$ 1.2, Ca^2+^ 2.35, Mg^2+^ 1.13, and glucose 5.0, in addition to 40% Dextran 70 in saline in order to maintain the colloid osmotic pressure in the absence of any protein (all compounds purchased from Sigma, UK). The perfusate was gassed with 95% O_2_ and 5% CO_2_, de-bubbled, pre-warmed to 37°C, and filtered with polymer wool before entering the plexus. During the experiment, the brain perfusion preparation was kept warm at 37°C in a water jacket, and by an external heat source. The composition of perfusion fluids in the aCSF contained in (millimolar): Na^+^ 148, K^+^ 2.9, Cl^−^ 135, ${\text{HCO}}_{3}^{-}$ 26, ${\text{HPO}}_{4}^{-}$ 0.25, Ca^2+^ 2.5, Mg^2+^ 1.8, and glucose 5.0. The aCSF was pre-warmed to 37°C, gassed with 5% CO_2_ in O_2_, and its pH adjusted to 7.2 prior to reaching the CPs. The perfusion pressure and brain temperature were continuously monitored by a pressure transducer and digital probe thermistor (Edal, CD model, UK). Under these experimental conditions, the brain preparation was viable for at least 5 h. A loss in viability was indicated by a rise in arterial pressure and a fall in the venous outflow.

### CSF Secretion Rate

This was determined from the difference in concentration of Evans blue albumin in arterial and venous perfusate samples. The concentration of the dye was determined using Unicam spectrophotometer at a wavelength of 625 nm. The secretion is given by Kf = Fv(*V*/*A*)-1 μl/min/g, where Fv = venous perfusion flow rate (μl/min/g wet weight), and *V* and *A* corresponds to venous and arterial spectrophotometer readings, respectively (59).

### Paired Tracer Indicator Dilution Technique

This technique was first developed to study the transport of sugar and amino acids across the isolated perfused CP of the sheep (59). We performed this technique in order to measure the uptake of ^125^I-labeled T_4_ from a bolus injection during a single circulation of the CP. This uptake of T_4_ is proportionally related to the passage of a non-transported marker molecule, ^14^C-mannitol. The uptake of [^125^I]-T_4_ (both net and maximum) was measured under conditions in which the isolated lateral CPs were perfused with different concentrations (50–200 µM) of unlabeled T_4_. Under these conditions, the 100-µl bolus contained both isotopes (labeled-[^125^I]-T_4_ and ^14^C-mannitol) in addition to different concentrations (50–200 µM) of unlabeled T_4_.

#### Experimental Procedure

A 100-µl bolus Ringer solution (perfusate) containing 3 μCi ^125^I-T_4_ and 1 μCi ^14^C mannitol was injected into a calibrated sidearm in the perfusion circuit and then switched into either left or right CPs *via* closed system of taps. After 25 s, the dead space within the tubing was cleared and then a run of 20 sequential “one drop” samples of venous effluent were collected in ~60 s, followed by continuous collection of one final sample during 4 min in order to calculate the flow rate. This was considered as 1 cycle of 21 samples per run, followed by collection of a clearance sample during 10 min. The above cycle was repeated for at least 4 times (*n* = 4–6) accounting for a total of at least 84 samples per brain. A 3.5 ml of scintillation liquid Ultima Gold (Packard, UK) was added to each of the 20 drops collected, as well as to the samples of the injected bollus. The activities of ^125^I and ^14^C in the samples were then counted and analyzed.

#### Calculation of the Recovered Isotopes

After counting the samples, the recovered ^125^I and ^14^C in each of the 20 drops was then expressed as a percentage of the ^125^I and ^14^C injected in the 100-µl bolus (% of injectate recovered). The following equation was used to calculate the percentage uptake (*U*%) for each drop, based on the differences in recovery of the two isotopes, taken into account that for any given drop the recovery of ^125^I-T_4_ from the CP is far less than the recovery of ^14^C-mannitol.

$$U\%=\frac{{\%}^{14}\text{C-mannitol recovered}-{\%}^{125}\text{I recovered}}{{\%}^{14}\text{C-mannitol recovered}}\times 100.$$

The net uptake *U*_net_ over the whole run was calculated from the single drops and the final “4 min” collection samples, as follows:

$$U_{\text{net}}\%=\frac{\sum {\;}^{14}\text{C-mannitol recovered}-\sum {\;}^{125}\text{I }{\text{T}}_{4}\text{ recovered}}{\sum {\;}^{14}\text{C-mannitol recovered}}\times 100,$$

where Σ is the sum of tracer recoveries for the whole run and the final “4-min” sample. Σ is expressed as percentage of the ^14^C or ^125^I originally injected.

### Steady-state Extraction at the Basolateral Face

This technique measures the extraction of ^125^I-T_4_ from the blood into CP over 1–2 h and was previously described (59, 60). The mammalian Ringer solution contained 10 μCi⋅100 ml^−1 125^I-T_4_ (90 pmol⋅l^−1^) and 40 μCi⋅100 ml^−1 14^C-mannitol as non-diffusible extracellular marker. The lateral CPs were perfused for 1 h until steady state has been achieved. The samples of arterial perfusate and venous effluent were collected at a regular intervals every 5 min, for a further 40 min, accounting for 8 samples per brain, for a total of 14 sheep. The tracer activities in 100-µl aliquots of arterial and venous samples were determined by liquid scintillation counting after addition of 3.5 ml of Ultima Gold (Packard, UK). The activities of both isotopes ^125^I and ^14^C were separated and converted to disintegration per minute (dpm); using internal stored quench curves on β-counter (LKB Rackbeta Spectral 1219, UK). The extractions of both ^125^I-T_4_ and ^14^C-mannitol at the blood side of the CP were calculated separately, using the equation below. The difference between the two extractions was considered as the cellular uptake of ^125^I-T_4_. Cellular uptake, also known as extraction (%) is

$$\text{Extraction }(\%)=\frac{F_{\text{a}}A^{\text{*}}-F_{\text{v}}V^{\text{*}}}{F_{\text{a}}A^{\text{*}}}\times 100,$$

where *F*_a_ = arterial flow rate (ml⋅min⋅g^−1^); *F*_v_ = venous flow rate (ml⋅min ⋅g^−1^ CPs wet weight); A*, V* = activity of the tracer ^125^I-T_4_ and ^14^C-mannitol in the arterial and venous effluent, respectively (dpm⋅ml^−1^).

### Statistics

All statistical calculations were performed using Microsoft Excel and GraphPad Prism version 5.0 (GraphPad Inc.). Results are expressed as the mean ± SEM. Statistical comparisons were performed using the Student’s *t*-test in order to determine statistical significance at *p* < 0.05. Symbols indicate statistical difference: *p < 0.05, **p < 0.001, ***p < 0.0001.

## Ethics Statement

The Institutional Animal Care and Use Committee (IACUC) of the Lebanese University approved all experimental procedures in this study. Surgical procedures were performed under deep anesthesia, and all animal experimental procedures were carried out in accordance with the guidelines of the Agriculture Ministry, which conforms to the provisions of the Declaration of Helsinki (as revised in Brazil in 2013) and of the European Communities Council Directive (86/609/EEC).

## Author Contributions

KZ and NK designed the study and performed experiments. NZ performed statistical analysis. MS, MH, WM, HH, and FK participated in data collection. KZ, FK, and NK analyzed data. KZ and NK wrote the manuscript. All the authors read and approved the final version of the manuscript.

## Conflict of Interest Statement

The authors declare that the research was conducted in the absence of any commercial or financial relationships that could be construed as a potential conflict of interest.

## Abbreviations

THs, thyroid hormones; T4, thyroxine; T3, triiodothyronine; CSF, cerebrospinal fluid; CP, choroid plexus; BBB, blood–brain barrier; B-CSF-B, blood–cerebrospinal fluid barrier; CNS, central nervous system; ECF, extracellular fluid; ISF, interstitial fluid; BCH, β-2-aminobicyclo-(2,2.1)-heptane-2-carboxylic acid.
